# Variability in aspirin efficacy: all in the genes?

**DOI:** 10.1093/eurheartj/ehz456

**Published:** 2019-07-05

**Authors:** Colin Baigent, Michael V Holmes

**Affiliations:** MRC Population Health Research Unit, Nuffield Department of Population Health, Oxford, UK

**This editorial refers to ‘Genetic variation at the coronary artery disease locus *GUCY1A3* modifies cardiovascular disease prevention effects of aspirin’^†^, by K.T. Hall *et al*., on page 3385.**

Meta-analyses of individual participant data from randomized trials have shown clearly that, when used for the secondary prevention of cardiovascular disease, the benefits of low-dose aspirin (75–100 mg daily) clearly outweigh the risks of bleeding,^1^ but the net benefit of aspirin for the primary prevention of cardiovascular disease is less clear,^2^ and has long been debated. The issue is topical, because last year three new trials assessing low-dose aspirin therapy (100 mg daily) vs. placebo for the primary prevention of atherosclerotic vascular disease (ASCVD) were reported: the ASPREE trial^3^ in elderly individuals, the ASCEND trial^4^ in patients with diabetes, and the ARRIVE trial^5^ in non-diabetic individuals with multiple risk factors for cardiovascular disease. An updated meta-analysis incorporating data from 13 primary prevention trials indicated that, even with the additional evidence provided by these three new trials, the absolute benefits of aspirin appear small, and are of a similar magnitude to the small absolute risks of major bleeding.^6^ It is unclear whether it is possible to identify people who might derive above average vascular benefit without also experiencing above average risks of bleeding. Since the relative risk reduction in serious vascular events (myocardial infarction, stroke, or vascular death) does not vary according to baseline prognostic factors,^2^ the absolute benefits of aspirin are proportional to the baseline risk of ASCVD. Therefore, it might be postulated that aspirin should be targeted at apparently healthy people at elevated ASCVD risk in whom the risks of bleeding are low. However, the risks of ASCVD and of bleeding are strongly correlated (chiefly because the risks of both outcomes are strongly determined by age), so few individuals are at increased risk of ASCVD without also being at higher risk of bleeding.^2^ Given the difficulty of selecting healthy individuals for aspirin use using conventional risk markers, it is of interest to consider whether subgroups defined by allelic variation might offer an alternative method of selecting people in whom the benefits of aspirin greatly exceed the bleeding risks.

Genome-wide association studies have shown that several chromosomal loci are associated with an increased risk of coronary artery disease^7^ and hypertension.^8^ One such variant is on chromosome 4q32.1, located within an intron and regulating expression of the *GUCY1A3* gene, which encodes the alpha-1-subunit of soluble guanylyl cyclase (sGC). One important mechanism through which platelet aggregation is inhibited *in vivo* is by endothelial production of nitric oxide (NO), which stimulates sGC to produce the second messenger cGMP, thereby inhibiting platelet aggregation (and promoting smooth muscle relaxation)^9^ (see *Figure 1A*). Patients homozygous for the common risk allele *GUCY1A3* rs7692387 (G) undergoing coronary intervention have been shown to display increased platelet reactivity.^10^ The authors of a new study reported in this issue of the *European Heart Journal* hypothesized that, because of this, individuals with the GG genotype may derive greater benefit from aspirin. They tested this hypothesis in genetic analyses of populations drawn from two randomized trials of aspirin vs. placebo in apparently healthy individuals.^11^

**Figure 1:**
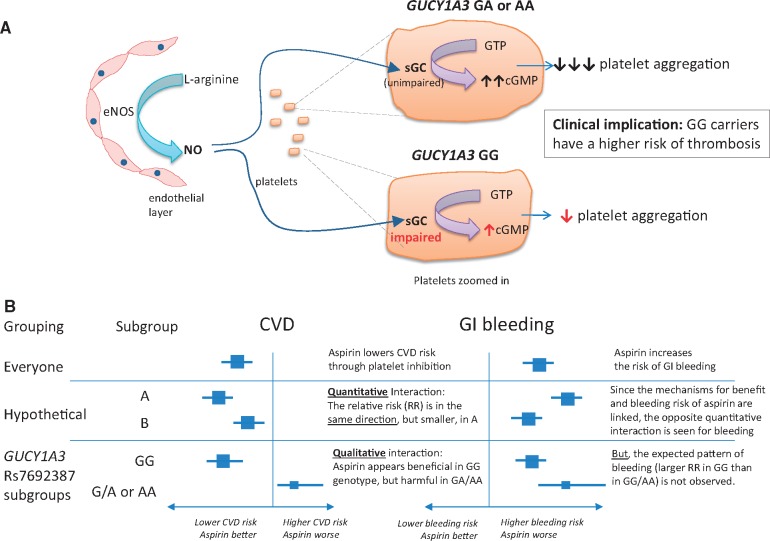
Postulated effects of *GUCY1A3* variants on platelet function. (*A*) Platelet response to nitric oxide; (*B*) relationship of genotype with response to aspirin on risk of cardiovascular disease and gastrointestinal bleeding. eNOS, endothelial nitric oxide synthase; NO, nitric oxide; sGC, soluble guanylyl cyclase.

The ‘hypothesis-generating’ sample included 23 294 women of European ancestry drawn from the Women’s Genome Health Study (WGHS).^12^ The WGHS was a genetic substudy in women who participated in the Women’s Health Study, a randomized trial of 39 876 healthy female healthcare professionals aged ≥45 years comparing aspirin 100 mg on alternate days vs. placebo (and, in a 2 × 2 factorial design, of vitamin E 600 IU vs. placebo).^13^ The validation (or ‘hypothesis-testing’) study included European men from case–control studies of myocardial infarction (MI) and of stroke embedded within the Physician’s Health Study (PHS) of 22 071 male physicians randomized to aspirin 325 mg on alternate days vs. placebo (and, in a 2 × 2 factorial design, to beta-carotene 50 mg vs. placebo).^14^

Estimates for the effects of aspirin in the WGHS were derived from subgroup analyses of the aspirin vs. placebo comparison among each genotypic category (GG, GA, or AA) that were designed to test for a treatment × genotype interaction. Since women were invited to participate in the WGHS prior to treatment allocation, such analyses would be expected to be unbiased with respect to aspirin assignment. In the PHS, however, the case–control structure did not allow for an analogous comparison of aspirin vs. placebo, so the effects of aspirin in each genotypic category were estimated using conditional logistic regression matched on age and smoking, and adjusted for randomized treatment assignment; as a consequence of this design, the effects of aspirin estimated within genotypic categories within the PHS may be subject to bias or confounding. The PHS therefore has limitations as a means of testing the hypothesis raised by the WGHS.

In the WGHS, there was an interaction between aspirin efficacy and *GUCY1A3* rs7692387 genotype (GG vs. GA/AA) such that aspirin appeared to reduce the risk of major cardiovascular events by 17% in women with the GG genotype (*P* = 0.08), but increased the risk by 39% in those with a GA or AA genotype (*P* = 0.03; Supplementary table S4 of Hall *et al*.). There was a similar pattern in the PHS sample, with a 37% reduction in the composite of MI or stroke in men with the GG genotype (*P* = 0.07), and a 32% increase in those with a GA or AA genotype (*P* = 0.47). It is of particular interest that the interaction observed in the WGHS was qualitative (i.e. aspirin yielded benefit in one subgroup and harm in the other), rather than quantitative (i.e there were differences in magnitude, but not the direction, of the effects of aspirin between subgroups); see *Figure 1B*. Such an interaction is extremely unusual, and immediately raises the question: are the findings biologically plausible?

An obvious way in which the plausibility of the results would be reinforced is if the observed effects of aspirin on gastrointestinal bleeding (or other measures of bleeding risk) exhibited the opposite pattern to that observed on ischaemic outcomes in each of the *GUCY1A3* genotypes (illustrated in the ‘hypothetical’ example in *Figure 1B*). This would be expected as both the anti-ischaemic effects of aspirin and its propensity to cause gastrointestinal bleeding result from the same mechanism (i.e. inhibition of prostaglandin synthesis in platelets, as well as in the gastroduodenum). In the WGHS, however, there was a quantitative, not a qualitative, interaction on gastrointestinal bleeding: aspirin increased gastrointestinal bleeding risk in both genotypes [the hazard ratios (HRs) and 95% confidence intervals were GG 1.10, 0.95–1.29; and GA 1.49, 1.19–1.86]. Consistency with the results on ischaemia would have required that aspirin caused a higher risk of bleeding in GG carriers than in GA carriers (indeed with the possibility that aspirin might even reduce the risk of gastrointestinal bleeding in GA carriers)—the opposite of the pattern that was actually observed. (Note that, while the HR for aspirin vs. placebo on gastrointestinal bleeding in the AA group was 0.85, there were only 29 cases of gastrointestinal bleeding in this category, and so there were too few events to calculate the HR precisely.)

It is well recognized that certain individuals may be resistant to the antiplatelet effects of aspirin, and many biological mechanisms have been proposed to explain such ‘aspirin resistance’.^15^ However, in this instance, an explanation for the observed results requires positing a mechanism by which the presence of a non-risk A allele for *GUCY1A3* rs7692387 leads not just to ‘aspirin resistance’ but to a phenotype in which aspirin induces a higher risk of thrombosis. Not only that, but the findings would imply that aspirin induces both a prothrombotic state and a bleeding diathesis among such individuals (who represent around one-third of the population). This seems to be inconsistent with our current understanding of the mechanism of action of aspirin, and is therefore somewhat implausible.

In the absence of a clear biological explanation for the counterintuitive findings, it remains relevant to ask why they might have emerged in the WGHS, and why they appear to be supported by the results of a second study (the PHS). Whilst this question cannot be answered with any certainty, it is worth pointing out that the observed interaction in WGHS on the effects of aspirin on major cardiovasular diseases between genotypic categories GG vs. GA/AA was not statistically extreme, and the ability of PHS to confirm the finding was constrained by the limitations of its case–control design. It remains possible that the findings were attributable to the play of chance. Therefore, they need to be replicated in other data sets, whether in the context of primary or secondary prevention, in which subgroup analyses of randomized comparisons of aspirin vs. placebo can evaluate reliably whether there are (directionally opposite) interactions between *GUCY1A3* genotypes and the effects of aspirin on ischaemic and bleeding outcomes. Until then, given the apparent incompatibility of these findings with what is known about platelet biology, the results of this study should be treated with caution.

**Conflict of interest:** C.B. reports grants from the Medical Research Council, UK, the British Heart Foundation, UK, and Boehringer Ingelheim, during the conduct of the study; and grants from Pfizer, Merck & Co., and Novartis outside the submitted work. M.V.H. reports collaborating with Boehringer Ingelheim in research, and fellowship funding from the British Heart Foundation, UK (FS/18/23/33512).

## Footnotes

^†^ doi:10.1093/eurheartj/ehz384.

## Notes

The opinions expressed in this article are not necessarily those of the Editors of the *European Heart Journal* or of the European Society of Cardiology.
